# Bringing your tools to CyVerse Discovery Environment using Docker

**DOI:** 10.12688/f1000research.8935.1

**Published:** 2016-06-21

**Authors:** Upendra Kumar Devisetty, Kathleen Kennedy, Paul Sarando, Nirav Merchant, Eric Lyons

**Affiliations:** 1 CyVerse, University of Arizona, Tucson, AR, 85721, USA

**Keywords:** CyVerse, virtualization platform, Discovery Environment

## Abstract

Docker has become a very popular container-based virtualization platform for software distribution that has revolutionized the way in which scientific software and software dependencies (software stacks) can be packaged, distributed, and deployed. Docker makes the complex and time-consuming installation procedures needed for scientific software a one-time process. Because it enables platform-independent installation, versioning of software environments, and easy redeployment and reproducibility, Docker is an ideal candidate for the deployment of identical software stacks on different compute environments such as XSEDE and Amazon AWS. CyVerse’s Discovery Environment also uses Docker for integrating its powerful, community-recommended software tools into CyVerse’s production environment for public use. This paper will help users bring their tools into CyVerse Discovery Environment (DE) which will not only allows users to integrate their tools with relative ease compared to the earlier method of tool deployment in DE but will also help users to share their apps with collaborators and release them for public use.

## Introduction

CyVerse (formerly iPlant Collaborative) ^1^ is the national life sciences cyberinfrastructure funded by the National Science Foundation (NSF). The infrastructure’s purpose is to scale science, domain experts and knowledge by providing a variety of computational tools, services, and platforms for storing, sharing, and analyzing large and diverse biological datasets. In addition, CyVerse provides a variety of resources to train scientists with diverse backgrounds to make the best use of CyVerse’s infrastructure and leverage advanced computational resources, including high-performance and cloud computing. The Discovery Environment (DE) in CyVerse provides a modern web interface for running powerful computing, data, and analysis applications. By providing a consistent user interface for accessing the tools and computing resources needed for specialized scientific analyses, the DE facilitates data exploration and scientific discovery. Because much of the complexity is hidden from the user, the DE makes it easy for non-technical users to run their analyses and for computational savvy users to share their apps with collaborators. Scientists do not need to master command-line analysis tools or learn new software for every type of analysis. All aspects of bioinformatics data management and analysis may be handled within the DE.

It is common in bioinformatics to build new analysis methods utilizing multiple programs, libraries, and modules, e.g., SAMtools or R with Bioconductor. However, each analysis that uses these tools requires specific versions of the operating system and underlying programs, such as Ubuntu version 14.04, Bioconductor version 3.2, R version 3.2.2, and SAMtools 1.3. In order to reproduce results, the same versions of software are often required, including supporting libraries and the underlying operating system. This delicate balance of dependencies, often called Dependency Hell, adversely impacts the reproducibility of analyses, and makes it challenging to share programs, workflows and analysis methods with collaborators and users who do not have access to identical systems. In the past, these issues have made it challenging for users to integrate new applications and analysis methods in the DE, as the underlying execution platform could only support a limited number of versions of the same software. For example, if your program expects to find BWA program version 0.7.12 ^2^ in /usr/local/bin, but another program expects version 0.7.13, it was impossible for the two versions to coexist without modifications to your code. With advances in container-based virtualization technology, these issues now are easily resolved, and support customized execution environments for every analysis.

Docker ^3^ is a container technology that wraps software of interest (e.g., a bioinformatics tool) together with all its software dependencies so it can run in a reproducible manner regardless of the environment. Compared to the previous method of tool integration in the DE, Docker images allow users to install multiple versions of software on the same system, streamlining the application integration process and ensuring that the final DE app will function as the user intended while developing it on their own compute platform (desktop, laptop or server). It also enables more complicated and difficult to install software (e.g., software with many additional dependencies) to be easily integrated in the DE. Even though containerizing applications has its own advantages, it does have certain limitations, such as limited access to large data or no web user interface. The DE helps solve some of these limitations by providing a web interface for integrated data management and tasks execution, while also streamlining the ability to upload, organize, edit, view, and share data and analysis with collaborators.

**Figure 1.**
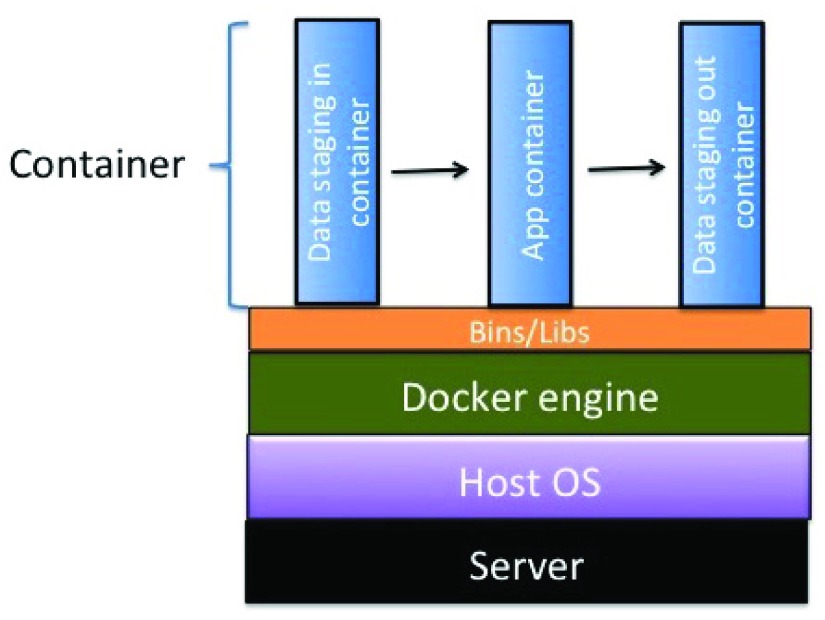
Container architecture in CyVerse DE.

CyVerse has adopted Docker for integrating software apps that run in the CyVerse DE’s Compute Cluster, which uses HTCondor for its resource-job-management system (RJMS). The user creates a Dockerfile, which is sent to CyVerse and used to build the Docker image containing the tool. After the image has been deployed on the DE’s compute cluster, the user can build an web app in the DE to use the tool. The Docker engine runs on three different containers ( Figure 1) in the DE:

- The data staging-in container delivers the data on which you want to operate from its location in the Data Store.
- The app container, based on your integrated Dockerized tool, runs with the data visible to it as a union file system.
- The data staging-out container returns data from the analysis that uses the app to the Data Store.

This compartmentalizes each major step of data movement and running analyses so that updates to each part can happen more easily.

## Methods

**Steps for Dockerizing a tool in the DE**: Before you can use a Dockerized image in DE, you must complete a few prerequisites:

- ***Install Docker and any other dependencies:***
  – Linux: The installation procedure involves the use of package containers, such as Curl, or the use of APT (Advanced Package Tool) and Yum repositories for your installation.
  – Mac OS X and Windows: Docker Toolbox is a quick and easy way to install and set up a Docker environment for Mac OS X and Windows.
  – Virtual Machine: Docker can be installed in a virtual machine environment through Virtual Box or Kitematic, which runs containers through a simple and powerful user interface.
- ***Ensure the tool you want to Dockerize is available from a reliable URL:***
  A reliable source is a website that hosts files/binaries necessary for tool installation and which can relied upon for future builds (e.g., ubuntu apt repos, redhat/centos yum repos, GitHub). An unreliable source is a public Dropbox link, a lab computer, a personal computer, etc.
  – If the tool and all its executables are available from reliable sources, use that URL for Dockerization of the tool.
  – If installation files cannot be retrieved from a reliable source, they should be version controlled with the Dockerfile by deposition in a code repository such as GitHub.

**Figure 2.**
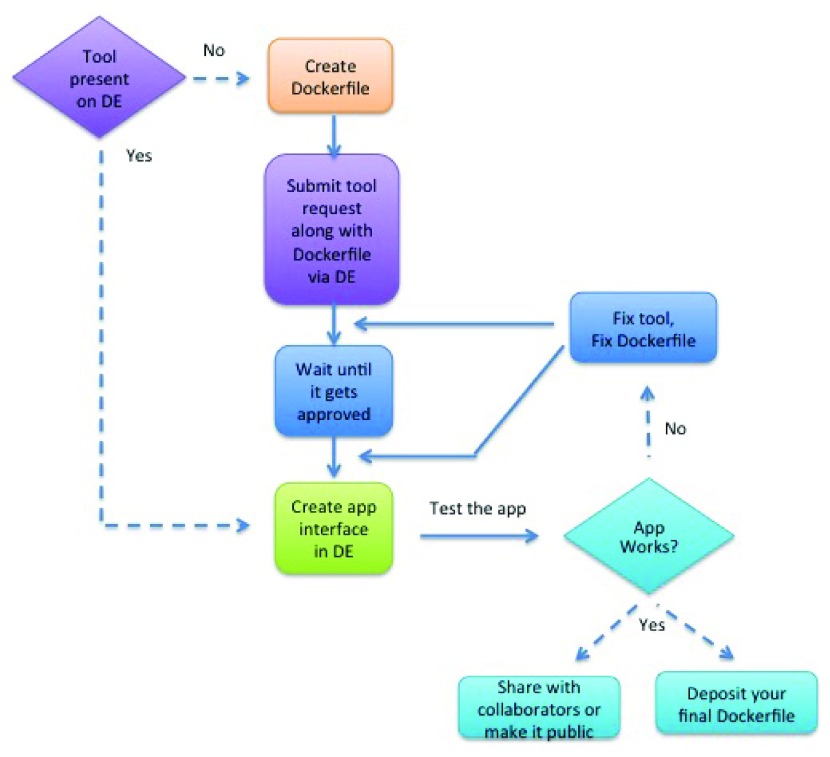
Flow diagram of Dockerization of tools in the DE.

The following steps ( Figure 2) along with this video tutorial serve as a guide for Dockerizing a tool in the DE.

**STEP 1: Check if the tool and correct version are already installed in the DE:** Before requesting installation of a new tool or new version of an existing tool, check the list of all tools in the DE to make sure that the tool and version you want is not already available:

1. Log in to the Discovery Environment by going to https://de.iplantcollaborative.org/de/, entering your CyVerse username and password, and clicking **LOGIN**. If you have not already done so, you will need to sign up for a CyVerse account. If you need to reset your password or forgot your CyVerse username, click **Need to reset your password?** and complete the form.
2. Click the **Apps** icon to open the Apps window.
3. Click the **Apps** menu item and then click **Create New**.
4. In the **Tool Used** field in the middle section, click the search icon to open the Installed Tools window.
5. In the search field, enter the first few letters of the tool name and then click the browse button, or scroll through the list until you find the tool to use.

All tools now run inside a Docker containers and are installed as Docker images in the DE. If the tool you want is not already available, you must first create a Dockerfile for the tool before requesting its installation in the DE.

**STEP 2: Create the Dockerfile** Construction of each tool’s image needs to be reviewed in order to ensure that it is reproducible and contains no security issues. The Dockerfile satisfies this goal of documenting how a tool and its software dependencies, including the base operating system, were installed. It is recommended that you adhere to the Docker community specific set of instructions. Additionally, follow these **CyVerse Dockerfile best practices**:

- Include all installation steps in the Dockerfile. For example, a Dockerfile should not copy and run a script that performs Ubuntu APT commands; instead, the APT commands should be in the Dockerfile.
- Write the Dockerfile to fail fast. This means that if anything goes wrong, construction of the image will fail immediately and the Docker image will not be deployed.
- Encapsulate the tool execution to avoid external dependencies unless by design (e.g., tool provides integration with external service).
- Derive the tool from an official Docker image (e.g., ubuntu:14.04.3 for the operating system).

**Sample Dockerfile: hisat2**—Dockerfile for installing hisat2 in a Docker container based on the Ubuntu:14.04.03 image:



                    FROM ubuntu:14.04.3
MAINTAINER Eric Lyons
RUN apt-get update && apt-get install -y \
    build-essential \
    git \
    python
ENV BINPATH /usr/bin
ENV SRCPATH /usr/src
ENV HISAT2GIT https://github.com/infphilo/hisat2.git
ENV HISAT2PATH $SRCPATH/hisat2
RUN mkdir -p $SRCPATH
WORKDIR $SRCPATH
RUN git clone "$HISAT2GIT" \
    && cd $HISAT2PATH \
    && git checkout 3f8c81375700d4107fdfd1caeaec01b5719ae4b8
RUN  make -C $HISAT2PATH \
    && cp $HISAT2PATH/hisat2 $BINPATH \
    && cp $HISAT2PATH/hisat2-* $BINPATH
ENTRYPOINT ["/usr/bin/hisat2"]
                


**STEP 3: Test the Dockerized tool** Before you request the installation of the Dockerized tool you should test the new image, using the docker run command:

                    docker run --rm <your/docker-image> <entrypoint arguments>
                


Your tool will most likely require inputs and produce outputs. If the tool’s image was built from a Dockerfile with an ENTRYPOINT and tagged with your/docker-image, then place some test input files into a scratch directory (e.g., ~/my-scratch-dir) and run a command like the following in that directory:

                    docker run --rm -v ~/my-scratch-dir:/working-dir -w /working-dir
your/docker-image user-input-1 user-input-2 ... 
                


The -v option mounts the current working directory on the host machine into the /working-dir directory inside the container. The -w option sets the working directory inside the container to that same /working-dir directory.

**Note:**

- The DE will run a tool’s Docker image using a combination of the docker run flags -w and -v" flags in order to mount the Condor node’s working directory to some arbitrary working directory inside the container. All inputs will be placed inside this working directory and the DE expects the tool to generate outputs in this working directory as well.
- Additionally, all arguments specified by the user in the DE’s app interface will be passed as command-line arguments to the docker run command following the your/docker-image name. From the example command above, these would be user-input-1 user-input-2 .... Exceptions are the Environment Variable fields, which will be passed to the docker run command as -e flags.
- Reference Genome/Sequence/Annotation input arguments are passed to the tool differently from other arguments, so if your tool requires these types of inputs, please inform the team of this requirement when you request installation of the tool.

If the tool’s container produced outputs in that host’s scratch directory, then this tool should be ready for the DE.

**STEP 4: Submit the request for installation of the Dockerized tool**

1. In the DE Apps window, click **Apps** and then click **Request Tool**.
2. In the Request New Tool Installation window:
  • Enter the name of the tool (executable or binary).
  • Enter a brief description of the tool as you want it to appear in the Apps window information section for apps that use this tool.
    – Enter the attribution for the tool, such as the person who created the tool (optional).
3. To submit your Dockerfile, in **How do you want to submit data for your tool’s source**, either:
  • Click **Enter a link** and enter or paste the URL.
  • Click **New upload** and browse to upload the file from your personal folder.
  • Click **Select from existing** and then browse to the source file location in the DE Data Store.
4. Enter the tool’s version.
5. Select the software architecture for the tool.
6. Specify if the file is multi-threaded. For information on threading, see Thread (computing) on the Wikipedia website.
7. If necessary, click to expand the **Other Information** section:
  • In the **How do you want to submit additional data** field, select either **New upload** (to upload the file to your personal Data folder), or **Select from existing** (to select a file that already exists in your personal Data folder), and then browse to select the first test data file. If choosing New upload, the filename must be unique.
  • Enter instructions for how to use the tool in the Unix environment.
8. To upload a second test data file, click **Browse** in the **How do you want to submit additional data** field and browse to select the second file (optional).
9. Enter any additional information that might be useful (optional).
10. Click **Submit**.

**Note:** Also note that Reference Genome/Sequence/Annotation input arguments are passed to the tool differently from other arguments, so if your tool requires these types of inputs, please include that requirement on the form when you request installation.

Your request is sent to CyVerse Support. When the new tool is installed, you will receive an email from CyVerse Support.

**STEP 5: Create and save the new app interface in the DE** Once the Dockerized tool is installed, you can create the DE app interface for the tool. The Create App window consists of four distinct sections ( Figure 4):

- The first section contains the different app items that can be added to your interface. To add an app item, select the one to use (hover over the object name for a brief description) and drag it into position in the middle section.
- The second section is the landing place for the objects you dragged and dropped from the left section, and it updates to display how the app will look when presented to a user.
- The third section (Details) displays all of the available properties for the selected item. As you customize the app in this section, the middle section updates dynamically so you can see how it will look and act.
- Finally, the fourth section at the bottom (Command line view) contains the command-line commands for the current item’s properties. As you update the properties in the Details section, the command-line view updates as well to let you make sure that you are passing the correct arguments in the correct order.

**Figure 3.**
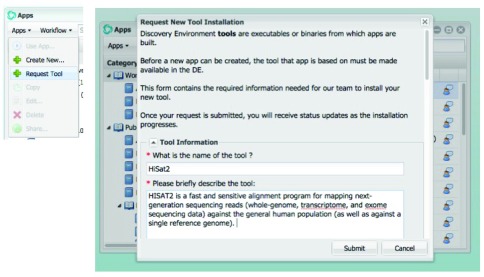
Request tool in DE.

**Figure 4.**
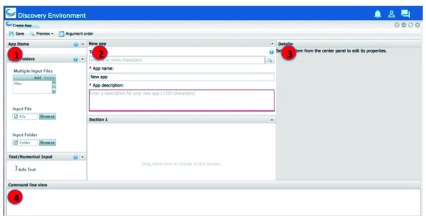
App user interface in DE.

Creating a new app interface requires that you know how to use the tool. With that knowledge, you create the interface according to how you want options to be displayed to a user. An app interface in the DE is arranged in a hierarchy of two main pieces:

- The framework consists of one or more *groups*. A group creates a conceptual boundary in the interface to organize options. For example, many DE apps have a first panel called File Inputs.
- Inside each group, you add those user interface objects you need to facilitate the collection of user inputs. There are a number of different user interface objects from which to select, including input file fields, selection and checkbox fields, text and numerical input fields, and output file fields.

**Figure 5.**
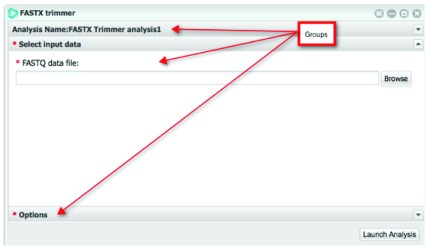
Designed DE app user interface.

In the example below ( Figure 5), we see three groups, with the **Select input data** group expanded.

At any step in creating or editing an app, you can preview the app and the its underlying JSON code. Previewing the app allows you to see how it will appear to a user, and to test how the app looks and functions. You also can preview and save the JSON code to a txt file.

Once you begin creating the app, it is highly recommended that you save your app frequently. Once saved, the app is available immediately in your workspace Apps under development folder (but not available to others to use).

**STEP 6: Test your app in the DE** After creating the new app according to your design, test your app in the your Apps under development folder in the DE to make sure it works properly.

- If your app works the way you expect it to, skip to Optional steps, below.
- If your app still needs a bit of work or if you need to make changes to your Dockerfile, go back to Step 2 and repeat.

**Optional steps:** Complete the additional steps as needed for your tool.

- **Editing an unshared app:** If you have not yet shared the app with the public (that is, it is still listed in your Apps under development folder in your personal workspace), you can modify its interface and create a new Dockerfile. If you create a new Dockerfile, email CyVerse Support to have it updated in the DE.
**Sharing (publishing) your app in the DE:** Once the app is working to your satisfaction and you have published it, you can share it with your collaborators or make it public for anyone to use. To share it with other users or make it public, see Sharing your App or Workflow and Editing the User Manual.
**Deleting/editing a publicly shared app:** After an app has been made public, it cannot be deleted because of CyVerse’s commitment to supporting reproducible science. You can, however, make a new version of the app and/or Dockerfile. Additionally, if you want to modify the app (e.g., expose more options in the interface), you can make a copy of the app and then modify that copy’s interface.
**Requesting a different category for your app:** When you share your app with the public, you will indicate the category or categories where it may be found. To request that your app be moved or added to a different or additional category, email CyVerse Support with the app name, current category or categories, and desired target category or categories.

## Use cases

Before you Dockerize a tool, it is important that you understand program dependencies (check the program documentation/manual thoroughly).

**Use case 1: Dockerizing a simple bioinformatics tool — Kallisto** The Kallisto Docker image was built on an Ubuntu-64 bit Virtual Machine using Virtual Box.

1. Install Docker (see the Methods section).

2. Create the Dockerfile.



                    FROM ubuntu:14.04.3
MAINTAINER Kapeel Chougule
LABEL Description="This image is used for running Kallisto RNA seq quantification tool"
RUN apt-get update && apt-get install -y build-essential cmake zlib1g-dev libhdf5-dev
RUN apt-get install --yes git
RUN git clone https://github.com/pachterlab/kallisto.git \
    && cd kallisto \
    && git checkout 5c5ee8a45d6afce65adf4ab18048b40d527fcf5c \
    && mkdir build \
    && cd build \
    && cmake .. \
    && make \
    && make install
ENTRYPOINT ["kallisto"]
                


3. Build the image.



                    Docker build -t"=ubuntu/kallisto" .
                


4. Test the built Kallisto image.



                    docker run --rm -v /Users/kchougul/Downloads:/kallisto_data -w kallisto_dataubuntu/kallisto kallisto index -i transcripts.idx transcripts.fasta.gz
                


**Use case 2: Dockerizing a bioinformatics tool - ParaAT** The ParaAT Docker image was built on Mac OS X using the Docker toolkit (quick start terminal).

1. Install Docker Toolbox for Mac OS X (see Methods section)

2. Create a paraAT git repo on GitHub

3. Create a paraAT git repo locally on your computer



                    git init paraAT
                


4. Clone the paraAT git repo to local



                    git clone https://github.com/jdebarry/paraat.git
                


5. Download the paraAT files, and then add and commit them to the local paraAT GitHub repo folder



                    git add . && git commit -m "adding paraat files"
                


6. Push the local paraAT repo to the remote repo



                    git push -u origin master
                


7. Create a Dockerfile



                    FROM ubuntu:14.04
MAINTAINER Jeremy DeBarry <jdebarry@cyverse.org>
# Get paraat.pl and epal2nal.pl code and add into $PATH to make it executable
anywhere, then make it executable
ADD https://raw.githubusercontent.com/jdebarry/paraat/master/ParaAT2.0/ParaAT.pl /usr/bin/
ADD https://raw.githubusercontent.com/jdebarry/paraat/master/ParaAT2.0/Epal2nal.pl /usr/bin/
RUN [ "chmod", "+x",  "/usr/bin/ParaAT.pl" ]
RUN [ "chmod", "+x",  "/usr/bin/Epal2nal.pl" ]
#Installing aligners, renaming clustalw executable so paraat.pl will use it -
this is a bandaid but it works
RUN echo "deb http://archive.ubuntu.com/ubuntu trusty multiverse" >> /etc/apt/sources.list
RUN DEBIAN_FRONTEND=noninteractive apt-get -qq update
RUN DEBIAN_FRONTEND=noninteractive apt-get install --no-install-recommends
-y clustalw mafft muscle t-coffee
RUN [ "mv", "/usr/bin/clustalw" , "usr/bin/clustalw2" ]
ENTRYPOINT ["ParaAT.pl"]
                


8. Build the paraAT image



                    docker build -t paraat .
                


9. Test the paraAT image



                    docker run --rm -v=/Users/jdebarry/Dropbox/Docker/paraat/Development/input/:/data paraat
-h /data/test.homologs -n /data/test.cds -a /data/test.pep -p proc -o
                


## Summary

The CyVerse Discovery Environment provides a simple yet powerful web portal for managing data, analyses, and workflows. The recent addition of using Docker to deploy new tools in the DE makes it easier and faster for users to incorporate and deploy their own tools in the DE with minimal effort. In addition, users can more easily share their tools, workflows, and knowledge with other researchers.

## Data and software availability

1. URL link to the Kallisto Dockerfile along with the test data - https://github.com/iPlantCollaborativeOpenSource/docker-builds/blob/master/kallisto/Dockerfile, doi: 10.5281/zenodo.53834 ^4^

2. URL link to the Paraat Dockerfile algon with the test data - https://github.com/jdebarry/paraat, doi: 10.5281/zenodo.53861 ^5^
