# Supplementary material for: Bringing your tools to CyVerse Discovery Environment using Docker
Source: F1000Res. 2016 Jun 21;5:1442. [Version 1] doi: 10.12688/f1000research.8935.1 (PMC5070597; doi:10.12688/f1000research.8935.1)
Supplement: Peer review history

# Peer review: Referee response for version 1

**DOI:** 10.5256/f1000research.9614.r16927

**Published:** 2016-11-14

**Authors:** Robert Cannon

**Affiliations:** 1 Textensor Limited, Edinburgh, UK

The authors present a clear description of how to use Docker to add tools to the CyVerse Discovery Environment. As a relatively recent development, Docker is having a major impact on the way many businesses deploy and update software for exactly the reasons described in the article. Clearly, the scientific use case is just as compelling in providing a way to deploy different tools on the same machine where they may have incompatible dependencies. 

 The steps for Dockerizing a tool and the various sample Dockerfiles provide a helpful guide to the whole process in the context of the CyVerse infrastructure.

I have read this submission. I believe that I have an appropriate level of expertise to confirm that it is of an acceptable scientific standard.

# Peer review: Referee response for version 1

**DOI:** 10.5256/f1000research.9614.r16925

**Published:** 2016-10-18

**Authors:** Thomas S. B. Schmidt

**Affiliations:** 1 Institute of Molecular Life Sciences, University of Zurich, Zürich, Switzerland

In this Software Tool Article, how the CyVerse Discovery Environment has been extended to integrate custom/user-provided (bioinformatics) applications using Docker. The article is clean and concise, contains several examples and downloadable data/code to recapitulate the described use cases. As an application note, the article provides an informative entry point for professional users and tool developers to get acquainted with CyVerse DE.

 In general, reproducibility is a very topical issue in various fields of life science research, and the use of approaches such as Docker-based environments present one way of tackling this. In this sense, the present article and the tool(s) it describes are timely and of use for the community.

 My only (minor) criticism is that in some parts the authors use jargon and overly technical language which might make the text less accessible to non-expert readers ("Dependency Hell", "BWA program", etc.). However, given that the main target audience are arguably bioinformatics tool developers, this is only a very minor point.

 Overall, I believe that the article and the work presented therein are a useful and constructive contribution to the field, and I am confident that the tools provided may greatly facilitate everyday research work for a wide range of potential users.

I have read this submission. I believe that I have an appropriate level of expertise to confirm that it is of an acceptable scientific standard.

# Peer review: Referee response for version 1

**DOI:** 10.5256/f1000research.9614.r14493

**Published:** 2016-07-01

**Authors:** Steven B Cannon

**Affiliations:** 1 Corn Insects and Crop Genetics Research Unit, United States Department of Agriculture, Ames, IA, USA

This Software Tool Article describes the way Docker has been incorporated into the CyVerse Discovery Environment. 

 The article makes a good case for use of Docker for reproducibility and to circumvent "dependency hell" and to enable more efficient sharing of complex software stacks.

 The article is clearly written, with nice examples and use cases.

 Points recommended for the review:

 - The title is appropriate.

 - The abstract provides an adequate summary of the article.

 - There is clear description of the use of Docker in the CyVerse DE environment, with clear examples and sample code.

I have read this submission. I believe that I have an appropriate level of expertise to confirm that it is of an acceptable scientific standard.
